# The current status and future prospects for molecular imaging-guided precision surgery

**DOI:** 10.1186/s40644-022-00482-2

**Published:** 2022-09-06

**Authors:** Imke Boekestijn, Matthias N. van Oosterom, Paolo Dell’Oglio, Floris H. P. van Velden, Martin Pool, Tobias Maurer, Daphne D. D. Rietbergen, Tessa Buckle, Fijs W. B. van Leeuwen

**Affiliations:** 1 Interventional Molecular Imaging Laboratory, Department of Radiology, Leiden University Medical Center, Leiden, the Netherlands; 2 Section of Nuclear Medicine, Department of Radiology, Leiden University Medical Center, Leiden, the Netherlands; 3 Department of Urology, ASST Grande Ospedale Metropolitano Niguarda, Milan, Italy; 4 Medical Physics, Department of Radiology , Leiden University Medical Center, Leiden, the Netherlands; 5 Department of Clinical Farmacy and Toxicology, Leiden University Medical Center, Leiden, the Netherlands; 6 Martini-Klinik Prostate Cancer Centre Hamburg, Hamburg, Germany

**Keywords:** Image-guided therapy, Intraoperative molecular imaging, Surgery, Fluorescence imaging, Multimodal imaging, Digital surgery, Surgical navigation, Pharmacokinetics

## Abstract

Molecular imaging technologies are increasingly used to diagnose, monitor, and guide treatment of i.e., cancer. In this review, the current status and future prospects of the use of molecular imaging as an instrument to help realize precision surgery is addressed with focus on the main components that form the conceptual basis of intraoperative molecular imaging. Paramount for successful interventions is the relevance and accessibility of surgical targets. In addition, selection of the correct combination of imaging agents and modalities is critical to visualize both microscopic and bulk disease sites with high affinity and specificity. In this context developments within engineering/imaging physics continue to drive the growth of image-guided surgery. Particularly important herein is enhancement of sensitivity through improved contrast and spatial resolution, features that are critical if sites of cancer involvement are not to be overlooked during surgery. By facilitating the connection between surgical planning and surgical execution, digital surgery technologies such as computer-aided visualization nicely complement these technologies. The complexity of image guidance, combined with the plurality of technologies that are becoming available, also drives the need for evaluation mechanisms that can objectively score the impact that technologies exert on the performance of healthcare professionals and outcome improvement for patients.

**Supplementary Information:**

The online version contains supplementary material available at 10.1186/s40644-022-00482-2.

## Introduction

Molecular imaging is increasingly being used to diagnose a range of diseases and to monitor and guide therapy, particularly in cancer. In this setting, molecular imaging often aids in the selection of systemic versus locoregional therapies [1]. The combination of diagnostics and therapy is commonly referred to as theranostics. In the nuclear medicine imaging literature, this approach typically refers to the application of radionuclide therapy based on demonstration of high expression of a therapeutic target presented on a diagnostic scan using PET or SPECT. However, a less-well recognized aspect of theranostics is the use of imaging to guide device-based interventions. Prime examples include percutaneous needle placement [2] and liver embolization [3]. These approaches are generally guided by imaging techniques such as ultrasound, or CT and SPECT ‘scout’ scans. Image -guided therapy equally applies to surgery, a field where precision planning can directly impact patient care. Image guidance can in particular be used to illuminate surgical targets (diseased tissue) or non-targets (healthy tissue that needs to be spared).

In surgery, favorable outcomes are achieved by excising all diseased tissue. At the same time the severity of complications is related to the invasiveness of an intervention. Hence, a balance needs to be created between the need for radical removal of disease and minimizing the scope of surgery. Accordingly, there is a drive towards minimal-invasive and more personalized interventions, while providing patients and healthcare professionals more confidence in the efficacy of radical resection, as well as uncomplicated postoperative recovery. From the very beginning of surgical practice decision-making has been guided by the tactile and visual senses of the operating surgeon. The technological advances made in the last century now allow these senses to be complemented via the use of preoperative imaging roadmaps (e.g., CT, MRI, SPECT and PET) and intraoperative target visualization in the form of white-light endoscopic video-image guidance. This evolution is helping minimally invasive ‘key-hole’ approaches to gradually replace open surgery. A clear example is the rise of robot-assisted laparoscopic surgery [4], which some now consider the standard-of-care in the resection of prostate cancer [5, 6]. The flip side of this minimally invasive trend is the loss of “touch” and thus a growing reliance on image guidance. An obvious next step in advancing image guidance in minimally invasive surgery is the inclusion of intraoperative molecular imaging strategies. Such strategies can help assist in target identification to guide resection of disease sites more accurately, while preserving delicate healthy anatomy and have led to the development of the concept of image-guided surgery [7, 8].

**Fig. 1:**
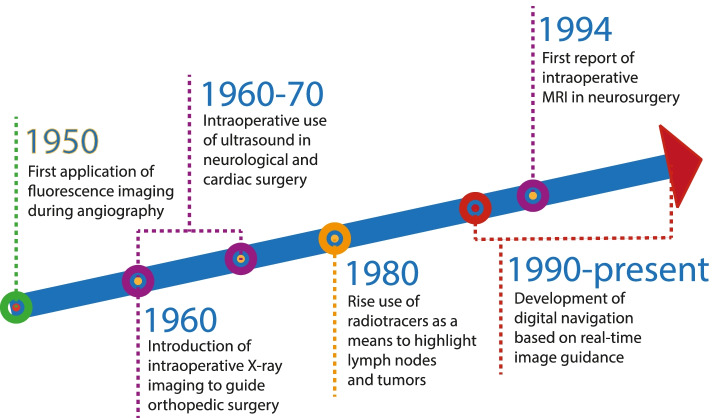
Timeline of the introduction of image-guided surgical technologies

Historically, image-guided surgery has been pursued using different modalities. Since the late 1950s fluorescence imaging has been implemented during angiography ([9–11]; Fig. 1). In the 1960s, intraoperative X-ray devices started to be used to provide imaging as guidance for orthopedic surgical interventions [12] and intraoperative ultrasound (US) was introduced to guide surgeons during neurological and cardiac surgery. The latter became more widely accepted in the late 1970s, mainly for application in general surgery [13]. Since the 1980s, radioactive tracers (radiotracers) have been implemented to highlight lymph nodes [14, 15], and later tumors [16]. Although scarce, there have even been reports describing the use of intraoperative magnetic resonance imaging during neurosurgery (first report in 1994, [17]). More exotic modalities are the use of magnetic particles [18, 19], optoacoustics [20], and Raman spectroscopy [21]. Digital navigation based on preoperative computed tomography (CT) or magnetic resonance imaging (MRI) roadmaps has been seen since the 1990’s with examples in head-and-neck, neuro- and orthopedic surgery [22]. Combined these approaches provide the foundations for evolving image-guided surgery.

The success of image-guided surgery is driven by the synergy between four main generic components: 1) relevance and accessibility of the target (medicine), 2) imaging agents (chemistry/pharmacology/pharmaceutics), 3) modalities used to detect or navigate towards the target defined in via imaging (engineering/imaging physics), and 4) interpretation of the imaging data (computer visualization). In this review, the status and future prospects of these four aspects of image-guided surgery is addressed. In addition, we indicate how each of the components can contribute to transition of new concepts from a laboratory setting into standard clinical care pathways (translational medicine).

## Results

### Target tissues

The concept of image-guided surgery has been most extensively pursued in the field of oncology. In this setting, molecular imaging has helped raise the diagnostic standard and increase the accuracy with which target tissues can be non-invasively identified. Key aspects herein are exemplified by the synergies of modalities such as positron emission tomography/computed tomography (PET/CT) with receptor-specific radiotracers such as [^68^ Ga]-octreotate, [^68^ Ga]-DOTATOC [23] and [^68^ Ga]-PSMA-11 [24]. Through this combination nuclear medicine has been able to demonstrate that receptor-mediated imaging based on tumor cell-related receptor overexpression allows accurate patient staging of lesions that are > 2 mm in diameter. Thereby enabling the identification of patients who may be suitable for surgical resection with curative intent.

**Fig. 2:**
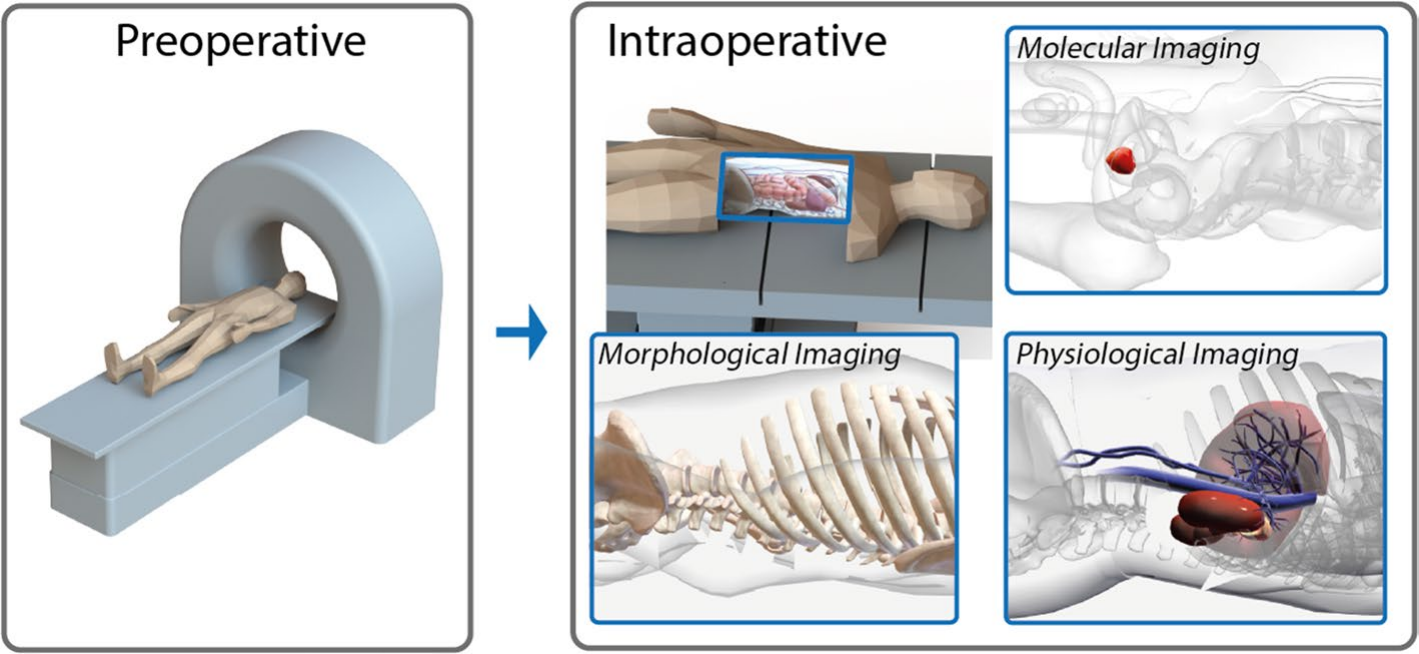
Schematic representation of the different types of imaging and their targeting principles. Preoperative imaging (radiology, nuclear medicine), morphological (anatomy), physiological (tissue level) and molecular imaging (cellular level) each suffer from a different signal attenuation, resulting in a different penetration depth

Image guidance is routinely used to clearly delineated organs such as blood vessels (angiography; indocyanine green (ICG; [25], US [26], X-ray [27]), bile ducts (ICG [28]), parathyroid (autofluorescence [29, 30]), lymph nodes (ICG and fluorescein [31, 32]), bony structures (X-ray; [33]), and macroscopic tumor lesions (US, 5-ALA; [34, 35], Fig. 2). Despite the widespread implementation of these applications the real promise of image guidance lies perhaps in indications wherein radical resection of microscopic or diffuse infiltrative lesions is required, with adequate safety margins. Unfortunately, application of image guidance in these indications also provides the toughest challenges as it requires a combination of tracers with high affinity and specificity for diseased tissue, and the generation of target to background contrast that allows accurate detection with the chosen instrumentation. An important drawback herein is that diffuse infiltrative cancers may directly translate into low signal intensities, limiting sensitivity for image guidance. The effect of signal intensity becomes even more important when realizing that during excision a safety margin in the range of 5–10 mm often has to be applied. This means that detection needs to be efficient through a substantial amount of tissue and therefore attenuation and scattering of signal are important considerations.

**Fig. 3:**
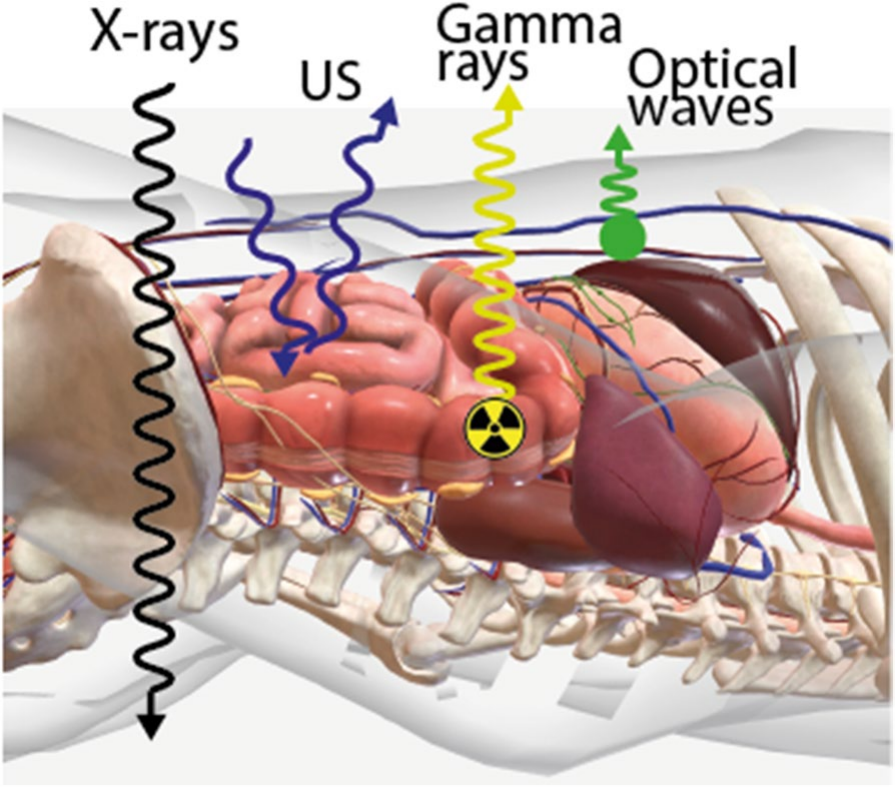
Tissue penetration of different imaging modalities. Each imaging modality uses another type of signal for image reconstruction e.g., X-rays, US, gamma rays and optical waves and therefore suffers from a different signal attenuation resulting in a different penetration capability of the resulting signal

From the perspective of having high signal intensity and low background, radiotracers are generally superior to optical tracers (including optoacoustics and lifetime imaging). With regard to having low signal attenuation and being subject to scattering, X-ray is the superior modality, followed by radiotracer-based detection, then US and lastly, optical tracers ([36, 37], Fig. 3). An important limitation for all optical approaches is that these are subjected to tissue-interactions during target illumination and/or signal emission; an effect that is smallest for optoacoustic applications [38]. Next to the issue of sensitivity, identification of small lesions requires an imaging modality with a high spatial resolution. Herein optical technologies, and in particular fluorescence, are superior compared to respectively US, radiotracer-based and X-ray detection.

 When looking at the practical implementation of the above-mentioned modalities, some basic principles can be deduced. First and foremost, all routine optical imaging indications remain confined to superficial assessment (e.g., endoscopic procedures of the mucosal surface of the bowel or airways), whereby the impact of light attenuation of the emitted signal and background light can be kept to a minimum. In addition, these applications tent to rely on relatively ‘simple’ chemicals. In a relatively high dose these agents help study physiological aspects such as vascular-, lymphatic-, and bile- flow [39]. Compared to optical imaging, US can help to increase the detection depth up to a certain degree and allows for real-time dynamic visualization. That said, this modality does not really facilitate molecular imaging beyond the targeting of receptors in the epithelium of blood vessels due to the lack of contrast extravasation. Optoacoustics, on the other hand, provides an interesting integration of the favorable optical high spatial resolution and US characteristics. However, for lesions located deeper below the tissue surface detection based on radioactive signals is preferred. This preference is not only driven by the modality’s signature to be able to penetrate overlaying tissue during the resection, but is also strengthened by the ability to non-invasively create a preoperative ‘imaging roadmap’ that accurately visualizes the distribution of the imaging agent (preferably in three-dimensions (3D, [40–42]). Such a roadmap allows the operating surgeon to only pursue a targeted resection when there is sufficient evidence that lesions are effectively identified. In addition, this preoperative roadmap helps to accurately locate (satellite) lesions that lie beyond traditional dissection templates [43]. While intraoperative CT [44] or MRI [45] also facilitate surgical planning via similar 3D roadmaps, the low contrast sensitivity of these modalities makes them less suitable for molecular imaging [46]. Hybrid approaches wherein the strengths of individual modalities are integrated could realise a best-of-both-worlds scenario [47, 48].

### Trends in imaging agents for surgery

The range of applications wherein interventional molecular imaging is having clinical impact is increasing through advances in medical chemistry and radiochemistry. In particular, there has been significant development in the design of optical- (e.g., fluorescence, Cherenkov, optoacoustics, Raman) and radioisotope-based (e.g., gamma rays and beta particles) agents [49, 50].

From a chemical perspective, most of the efforts towards designing disease specific imaging agents find their origins in nuclear medicine and its subdiscipline of radiochemistry. Radioguided surgery applications for sentinel nodes (radiocolloids, [51]), somatostatin receptor overexpressing lesions (peptides, [52, 53]) and prostate specific membrane antigen (PSMA) expressing lesions (small molecules, [54]) have established ^99m^Tc (140 keV) and, to a lesser extent, ^111^In (gamma rays with photon energies of 171 and 245 keV) as the most favorable radioisotopes [55] for clinical use. This is primarily driven by the common availability of ^99^Mo/^99m^Tc generators in clinics world-wide and ^111^In being an accessible long-lived reactor product. This further focused tracer design, with recent examples of widely implemented agents being ^99m^Tc-PSMA-I&S [56], (ICG-)^99m^Tc-nanocolloid [48] and ^111^In-octreotide [57]. Besides the application-specific design of radiotracers there are various attempts to use off-the-shelve PET tracers for image guidance by exploring i.e., their 511 keV gamma rays [58], beta particles [59] and/or Cherenkov light [60]. Advantages of the use of radiotracers are that they can be applied under a micro-dosing regime, are compatible with quantitative biodistribution studies (%ID/g) and support non-invasive pre-operative imaging approaches such as scintigraphy, single photon emission computed tomography (SPECT) and PET (Table 1).

**Table 1:** Examples of radio-, fluorescent and hybrid tracers used for preoperative imaging and intraoperative guidance

	**Preoperative imaging**	**Intraoperative guidance**	**Hybrid tracer**
Blood flow	Gd-DTPA (MRI; [61]), Iomeron (CT; [62]), Optison (US; [63])	ICG (fluorescence; [64]), Fluorescein (fluorescence; [65])	-
Sentinel lymph nodes	Radiocolloids ^*^ (SPECT; [66])	Radiocolloids ^*^ (γ probe; [66]), SPIONs (magnetic probe; [67]), ICG (fluorescence; [66]), Fluorescein (fluorescence; [68])	ICG-^99m^Tc-nanocolloid (γ probe and fluorescence; [66])
Biliary excretion	^99m^Tc-mebrofenin (SPECT; [69]), ^99m^Tc-disofenin (SPECT; [70])	ICG (fluorescence; [71])	-
(Para)thyroid	^123/131^I-pertechnate (SPECT; [72]), ^99m^Tc-sestamibi (SPECT;[73]), Iodine (SPECT; [74])	ICG (fluorescence; [75]), ^123/131^I-pertechnetate (γ probe; [76]), ^99m^Tc-sestamibi (γ probe; [77]), autofluorescence (fluorescence), Iodine (γ probe; [77])	^124^I (Beta-probe, Cerenkov imaging; [55]), ^123^I‐Methylene Blue (γ probe visual blue; [77])
Metabolism	^18^F-FDG (PET; [55]), ^123/131^I-MIBG (SPECT; [78])	5-ALA (PpIX; fluorescence; [79–81]), ^125^I-MIBG (γ probe; [82])	-
Receptor targeted			
Prostate cancer (PSMA)	^68^ Ga-PSMA (PET; [83]), ^18^F-PSMA (PET; [84]), ^99m^Tc-PSMA-I&S (SPECT; [40])	^68^ Ga-PSMA (beta-probe; [83]), ^99m^Tc-PSMA I&S (γ probe; [40])	^68^ Ga-PSMA (beta-probe and Cerenkov; [85]), ^68^ Ga-Glu-urea-Lys-(HE)_3_-HBED-CC-IRDye800CW (PET and fluorescence; [86])
Somatostatin	^68^ Ga-DOTATOC (PET; [87, 88]), ^111^In-octreotide (SPECT; [89]), ^99m^Tc-deptreotide (SPECT; [90]), ^68^ Ga-DOTATATE (PET; [91])	^68^ Ga-DOTATOC (beta probe; [55]), ^111^In-octreotide (γ probe; [53]), ^99m^Tc-deptreotide (γ probe; [53])	-
Tyrosine-protein kinase Met (C-Met)	^68^ Ga-EMP-100 (PET; [92])	EMI-137 (fluorescence; [93, 94])	-
Integrins (a_V_b_3_)	^68^ Ga-RGD (PET; [95]), ^18^F-RGD (PET; [96])	cRGD-ZW800-1 (fluorescence; [97])	^124^I‐cRGDY‐PEG‐C (beta probe, fluorescence; [98])
Vascular endothelial growth factor (VEGF)	^89^Zr-Bevacizumab (PET; [99])	CW800-Bevacizumab (fluorescence; [100]),	-
Human epidermal growth factor-2 (Her-2)	^68^ Ga-Her2 (PET; [101])	CW800-HER2 (fluorescence; [102])	^111^In-HER2-IRDye800CW (γ probe, fluorescence; [103])
Carcinoembryonic Antigen (CEA)	^111^In-DTPA (SPECT; [104]), ^111^In-IMP288 (SPECT; [105])	FITC‐CEA mAb (fluorescence; [106]), SGM-101 (fluorescence; [107])	^111^In-DTPA-SGM-101) (γ probe, fluorescence; [104]), FITC‐^125^I‐ CEA mAb (γ probe, fluorescence; [108])
Epidermal growth factor receptor (EGFR)	^89^Zr-Cetuximab (PET; [109])	cetuximab-IRDye800 (fluorescence; [110–112]), panitumumab-IRDye800 (fluorescence; [113])	-
Carbonic Anhydrase IX	^89^Zr-Girentuximab (PET; [114]), ^124^I-redectane (PET; [115])	-	^111^In‐DOTA‐ Girentuximab-IRDye800CW (γ probe, fluorescence; [116])

^*^ e.g. ^99m^Tc-(nanocolloid, Senti-Scint, phytate colloid, tin colloid, sulfur colloid)

**Fig. 4:**
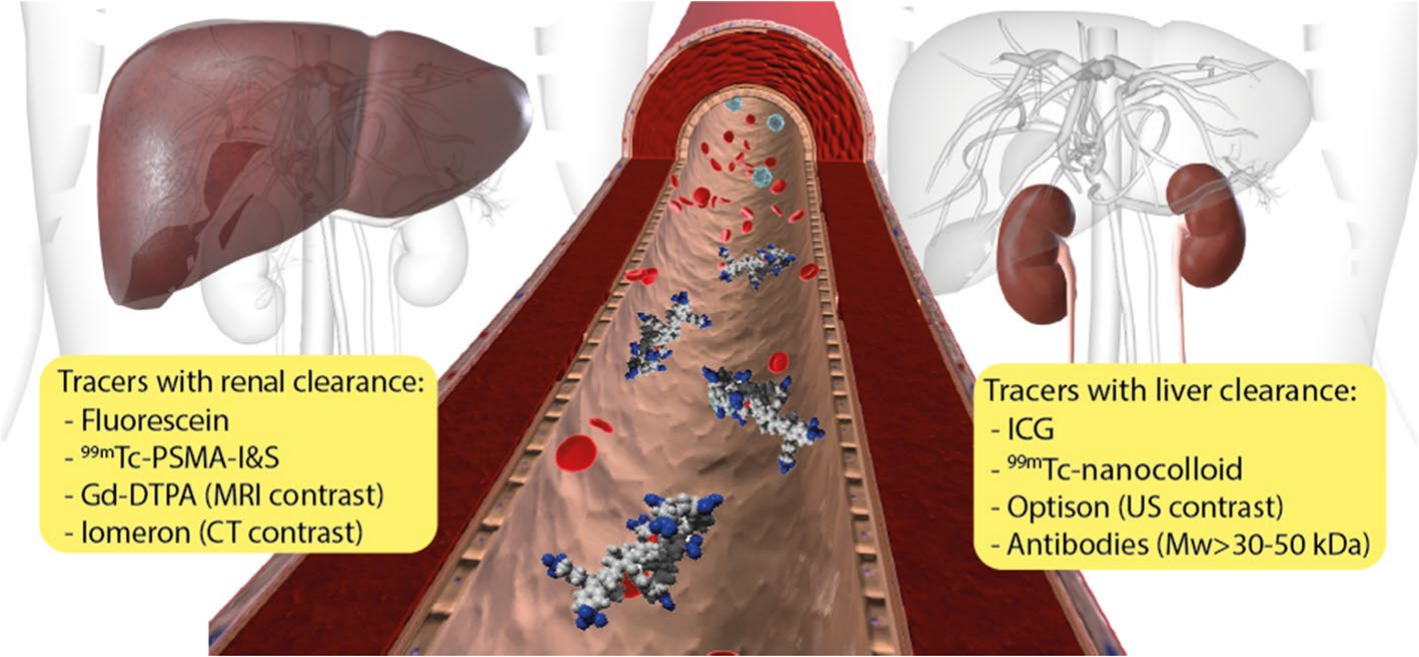
Tracer clearance. Examples of contrast/ imaging agents that are either excreted through the liver or through the renal system depending on their chemical and pharmacokinetic characteristics

Second in popularity is the development of fluorescent tracers intended for superficial lesion identification. Where fluorescence microscopy tends to focus on use of dyes in the 400-700 nm range, fluorescence-guided surgery efforts often tend to explore the theoretically favorable interaction between near-infrared (NIR) fluorescence (> 750 nm) and tissue [117]. NIR wavelengths are said to allow deeper penetration depth without visual obstruction of the surgical field caused by the dye. Interestingly, there is mounting evidence that fluorescence emissions outside of the NIR range equally hold promise for in-human use [118], with a prime example being the FDA-approved use of 5-ALA in neurosurgery [79]. While fluorescence imaging cannot be used to obtain preinterventional roadmaps, fluorescent agents are increasingly used in combination with some form of diagnostic nuclear medicine scan (see Table 1 for examples). This concept is most valid when the fluorescent and nuclear agents have the same target affinity, pharmacokinetics and can be applied in the same dosing regimen. Unfortunately, the molecular properties of the relatively ‘bulky’ fluorescent labels can exert a substantial influence on the affinity and pharmacokinetics of small-molecule and peptide-based tracers [119]. There is even literature suggesting that fluorescent dyes may alter the pharmacokinetics of ‘large’ nanobodies [120] and antibodies [121]. An additional downside of fluorescent agents is that their biodistribution cannot be assessed quantitatively based on fluorescence intensity alone, due to autofluorescence, signal scattering and limited tissue penetration of the signal. Moreover, in contradiction to the low administered mass of radiotracers and subsequent lack of biological effects with most agents [122], fluorescent tracers tend to be used at pharmacologically-active dose levels [123]. Use of such high dose levels may reduce the number of false negative results but is also likely to increase non-specific (background) staining. This enhances the number of both false negatives (due to loss of contrast) and false positive results. Hereby is it must be noted that fluorescent dye properties such as lipophilicity, charges and level of serum binding influence the pharmacokinetics, in particular the mechanism of clearance (Fig. 4). For obvious reasons the effect of the latter can be quite critical when the surgical target is located directly within (e.g., kidney or liver) or immediately downstream of the renal of hepatobiliary clearance route (e.g., prostate or bowel) where unbound excreted tracer can severely hamper lesion identification.

A strategy to overcome the limitations of individual modalities is the use of bimodal/hybrid imaging agents. Herein, nuclear medicine signals tend to be complemented with optical [124], magnetic [125], or US [126] contrast. In particular, nuclear/optical applications have demonstrated value in the surgical setting [50]. Combining two signatures in a single imaging agent allows for detection by two independent modalities, thereby supporting all relevant aspects in pre- and intraoperative imaging. As can be derived from the above, such hybrid agents will have different detection sensitivities for the different signatures. Ideally, the fluorescence sensitivity of hybrid agents is improved. However, the means to do so are limited. Hereby it is important to note that self-quenching of fluorescent dyes occur when dyes that reside on the same molecule are located within 8 nm of each other [127, 128]. For most imaging agents this means that there is an optimum in the number of dyes used as label e.g., 1 to 1 molar ratio [129]. This essentially means that improvements can only be realized by tuning the fluorescence brightness of dyes (a multiplication between i.e., molar extinction coefficient and the quantum yield). The most common dyes used in image-guided surgery applications are cyanine dyes, wherein extension of the length of the -C = C- bridge facilitates the use of higher wavelengths, but at the same yields suboptimal trans-confirmations [130]. As a result analogues of the cyanine dye Cy7.5 such as the commonly used ICG have a low brightness [131]. Introduction of charged moieties e.g. -SO_3_^−^ has been shown to enhance the brightness [132].

### Trends in medical devices for interventional imaging

The ideal intraoperative detection modality would probably be best described as a device that: 1) has a high sensitivity for signal detection, 2) only marginally suffers from interference by non-specific background signal (high specificity) and 3) maintains or improves present surgical logistics. These are generic wishes that transcend across all modalities.

It is no surprise that the imaging physics drive the design of a medical device used for interventional imaging. Conventional X-ray approaches such as CT and fluoroscopy are often impractical for implementation in the surgical suite, and accordingly, surgical interventions more often implement X-ray imaging in the form of a c-arm design [133]. In some cases, even C-arms prove to be incompatible with the surgical setup [134]. US requires a relatively small transducer that contains an integrated pulsed sound source and detector capable of registering reflected sound waves [135]. As air interferes with the detection, the transducer needs to be placed in direct contact to tissue, often requiring the use of conductive gel. Optical technologies such as fluorescence imaging does not require direct contact with tissue for a light source to excite fluorophore molecules and a detector to collect the light that is subsequently emitted [136]. Since light sources including ambient light [137], plenum light [138] and light emitted by optical tracking systems [139] can interfere with signal detection, fluorescence applications often require dimming of interfering light sources such as the operating room (OR) lights. Radioguidance modalities are purely designed to detect radiopharmaceuticals that intrinsically generate a signal (i.e., gamma rays or beta particles). However, to determine the position of the emission source within the patient (i.e., the radiopharmaceutical), collimation is required. Interestingly, surgical modalities have also been combined in hybrid, or multimodal, devices. Examples being: a C-arm with integrated gamma detector [140], gamma detector with integrated fluorescence imaging [141], several versions of SPECT or gamma detection integrated with US [142, 143] and beta detection integrated with optical coherence tomography [144].

**Fig. 5:**
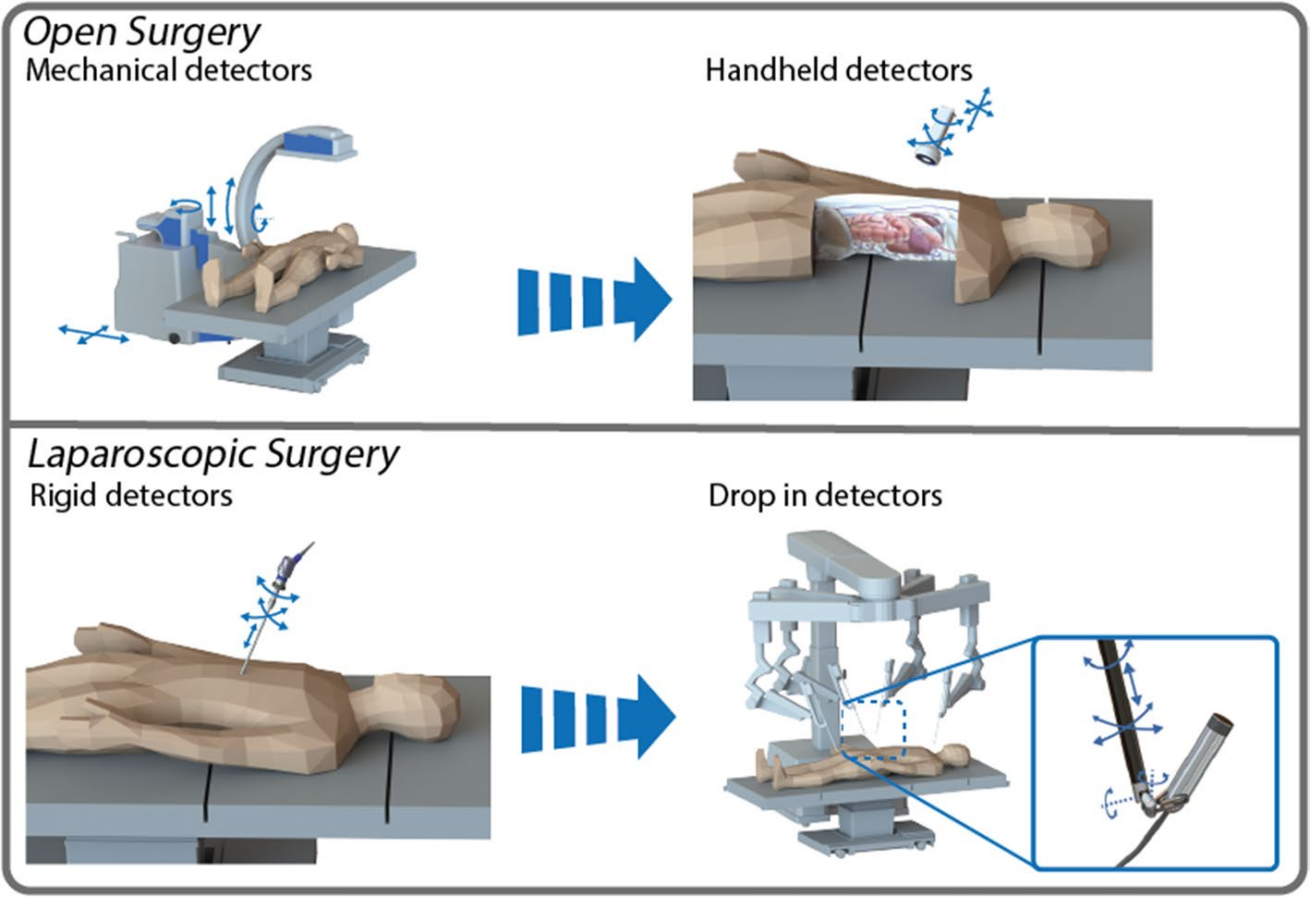
Detectors in open and laparoscopic surgery. The various kind of intraoperative detectors used for image guided surgery (open and laparoscopic) including their movement’s degrees of freedom

Effective application of imaging modalities during surgery is highly dependent on the degrees of freedom with which the modality can be positioned relative to the target. In open surgery (Fig. 5), cameras are not necessarily restricted in size other than the footprint that they occupy in the OR and the investment costs. Handheld probes (gamma [145], fluorescence [146], US, and magnetic [147]) as well as mobile gamma and fluorescence cameras [148] set the standard today (Fig. 5). The designs of these modalities can vary substantially. For example, probes are often provided in different detection angles (e.g., 0°, 45° and 90°) and mobile cameras are available as handheld device (e.g., Crystal cam [149], PDE-neo II [148]) or attached to a mechanical positioning arm (e.g., Sentinella or VITOM). Detection angles and size reductions can facilitate the accessibility of certain restricted anatomies, while mechanical arms offer stable positioning but often result in loss of dynamic flexibility. Taking fluorescence-guided surgery systems as an example, enlargement of detectors and excitation light sources can provide a boost in sensitivity [148]. This is a feature that may substantially increase utility, but also comes with increases in cost. While common practice, improvements in sensitivity do not necessarily result in an enlarged footprint, as they may also come from improved detector materials and refined signal processing [106, 138].

A stated earlier surgery is increasingly performed in a minimally invasive setting (Fig. 5, laparoscopic surgery). While the signals that need to be detected tend to stay the same, this change in environment requires a substantial change in the design of an imaging modality. The main driving factor herein can be attributed to the physical restrictions that are inherent in “key-hole surgery”, meaning that the entry point limits both instrument size and movements [150, 151]. Interestingly, minimally invasive interventions tend to go hand-in-hand with the loss of tactile sensing and thus, increasing the demand for ‘molecular-sensing’ technologies. For such modalities miniaturization is the focus of the current general design trend. In most instances this translates to a loss in sensitivity when compared to the open surgery setting. A key example herein is fluorescence guidance [152]. Uniquely for radioguided surgery the gamma-detectors used in probes remain similar for both the open and laparoscopic devices, thus preserving sensitivity [150]. Following the design of laparoscopic surgical instruments there is a trend to move from ‘rigid’ laparoscopic modalities to ‘steerable’ ones. Examples are the use of tethered drop-in detectors for US [153, 154], gamma-tracing [155], beta-tracing [156].

### Trends in digital surgery

**Fig. 6:**
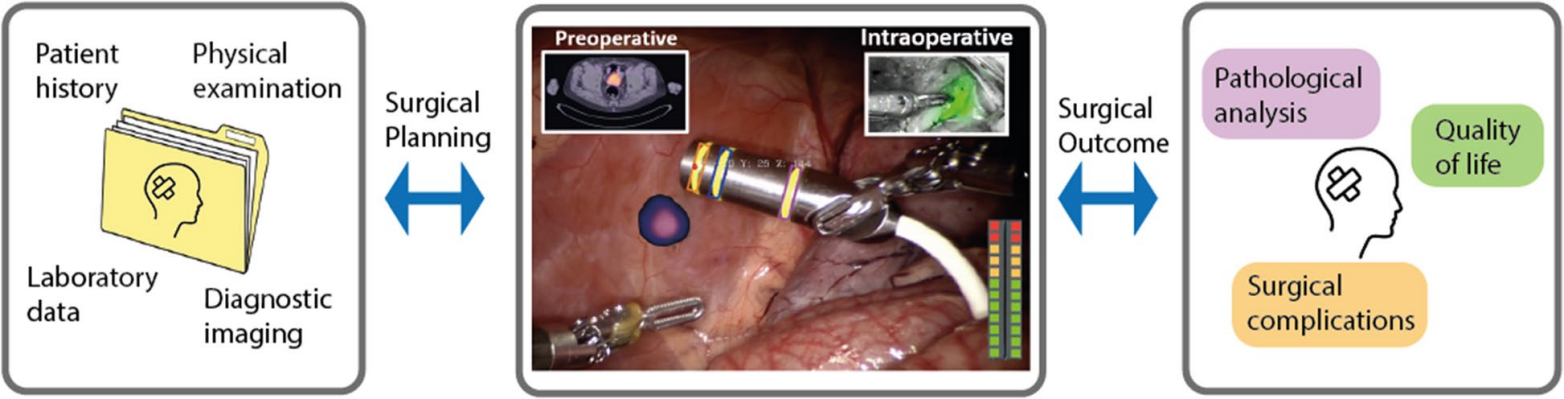
Schematic explanation of the workflow in a digitally enriched surgery. Starting with the input of patient data for surgical planning. Followed by the execution of the digitally enriched surgery, including input of preoperative and intraoperative scans, tool tracking and navigation towards the target. Afterwards the surgical outcome is assessed on pathological analysis, surgical complications and if/how much the quality of life has been impacted

The use of target-specific contrast agents and advanced interventional modalities aligns nicely with the promising new sub-discipline of digital surgery. The concept behind digital surgery joins the power of robotics, world class instrumentation, advanced imaging and visualization, data and analytics. One may argue that an ideal procedural work flow would constitute of: 1) preoperative target assessment, 2) intraoperative navigation towards the target, and 3) intraoperative confirmation of the target location and margins [157]. A way to realize integration between these elements is through the digitization of the signals and the use of dedicated algorithms to align and interpret complementary outputs (Fig. 6).

One key aspect is the direct registration of preoperative imaging information onto the surgical field in the OR. Here, registration can take place based on endogenous structures or exogenous fiducials (also called markers). The most straight forward implementation of such registration concepts is surgical navigation, meaning that the operating surgeon can position the surgical instruments in the geographic context of preoperative images [158]. This approach is thoroughly embedded in interventions on rigid anatomies, e.g., orthopedic-, neuro- and head-and-neck surgery [22, 159, 160]. Navigation is used, for example, to guide the placement of patient tailored 3D-printed prostheses in pelvic reconstructive surgery [161]. In recent years, the concept of intraoperative navigation has been extended to soft-tissue interventions [162, 163]. However, in these soft-tissue applications real-time intraoperative imaging and therefore other modalities, such as US [164, 165], gamma probes [166] and/or fluorescence imaging [167, 168], are required to confirm the accuracy of the intraoperative navigation process.

Alternative to registration of preoperative images to the patient, intraoperative tracking and interventional modalities using exogenous fiducials, can help register the output of an imaging modality to a specific anatomical location in the patient; a concept used to generate so-called freehand scans. Freehand options have been reported for US [169], beta particles [170], gamma-ray [171], fluorescence [172], and magnetic signals [173], and have been utilized in both open and laparoscopic/robotic surgery [174]. Unique for open surgery applications is that larger detectors can be used (e.g., hand-held gamma cameras [175]) and that the rotational movement in general allows for better coverage. Uniquely, the use of a ‘drop-in’ gamma probe has allowed for freehand SPECT to be performed using a robotic platform [176]. A big advantage of freehand scans is that they make an overview of the situation that is encountered in the surgical setting. A downside, however, is that such scans are generally lower in quality and are often analyzed by the surgeons rather than expert radiologists or nuclear medicine physicians.

Another, perhaps more obvious, aspect of digital surgery is related to computer visualization applications. Such techniques help improve the interpretation of imaging data [177]. Intraoperatively, use of computer visualization predominantly extends the use of imaging modalities such as CT, US, gamma- and fluorescence cameras. Hereby dedicated algorithms can help enhance feature extraction and/or interpretation. A key example is the visualization of (NIR) fluorescence signals in artificial colors to improve contrast: white [178], blue [179], pink [180], or as rainbow coloration [181] whereby green (color for which the human eye is most sensitive) has been most abundantly used. Alternatively, signal intensities can be boosted digitally, where again examples can be found in fluorescence imaging [106]. A more advanced version of computer visualization is automated feature extraction and data quantification. Feature extraction can help simplify surgical and/or pathological tissue interpretations [182], but at the same time can be used to drive kinematic assessments of instrument movements [183].

While application of artificial intelligence in the realm of image-guided surgery is still limited [184], it is highly likely that such efforts will intensify in the future. In fact, this is an area where we can expect a significant impact over the coming years.

## Future prospects

Clinical translation of novel image-guided surgery technologies beyond research and development requires establishment of an evidence-base that demonstrates the safety and effectiveness of these procedures, while reimbursement requires evaluation of cost-effectiveness. Strictly speaking, image-guided surgery technologies need to provide either better outcome for the patient or improve the workflow for medical professionals, if not both, and at a reasonable cost. In this regard, patient benefit can be scored by looking at short-term complication rates and long-term outcomes across cohorts with and without use of image guidance. Unfortunately, availability of such data is limited as most studies seem to focus on proof-of-principle studies. Exceptions to the rule are represented by reports for 5-ALA [185], sentinel node procedures in melanoma patients [186], ICG-^99m^Tc-nanocolloid [48, 187, 188], and ^99m^Tc-PSMA-I&S [189, 190]. Assessment of the impact of implementing image-guidance on surgical procedural performance, is traditionally performed via use of qualitative questionnaires and the recording of surgical time [191]. Conceptually, it is challenging to make objective and quantitative assessments regarding the adequacy of surgery or a surgeon’s performance, since many individual factors may impact outcomes. Recently, as result of the performance-guided surgery paradigm, kinematic assessments have been put forward as a means to score the proficiency of a surgeon [192]. This approach has the potential to provide short term means to define the added value of an image-guidance technology [183]. Uniquely, assessment of the surgeon’s performance based on kinematics will not only allow study of how chemical and engineering efforts enhance the surgical experience but will also allow assessment of how these approaches complement each other. Ultimately such assessments can be related to outcome data.

As image-guided surgery solutions are quite often technically challenging. In essence, ethics and regulations provide a healthy translational hurdle to protect patients, while financial aspects may also constrain development. from a practical perspective, lead compounds and detection device prototypes must be developed and refined in research setting and often not within the domain of patient care. While this helps preventing the patient exposure to potentially harmful technologies, it also means that some approaches can become more ‘technology-driven’ than ‘clinical-need-driven’. Ideally all developments should be done with clinical translation in mind and based on real-life unmet surgical needs. But even with these prerequisites, it is extremely challenging to translate laboratory findings to the clinic. An example of the challenges faced is the extrapolation of findings in small animal disease-models to patients. Besides the obvious anatomical differences, which will reflect on tracer pharmacokinetics, the performance characteristics of modalities employed in small animals are not necessarily recapitulated in human instrumentation [50]. Regularly, images depicting surgery in mice that claim translational potential are published, but one should perhaps approach such claims with healthy skepticism. Phantoms can provide a size matched intermediate to test modalities on, but at the same time these settings are even more artificial than small animal disease models. This leaves large animal models like those used for surgical training purposes [180] as prime candidates for assessment of clinical potential of novel intraoperative imaging approaches. In large animals, the same type of tracers, modalities or software solutions can be used as are used in patients (note: devices used on animals can no longer be used for clinical purposes). These can then also be validated in a surgical environment that is close to the intended use. A limitation is that large animal surgical-training-models are less suited as disease models as creation of such models is a costly and time-consuming ordeal that raises several ethical issues. An approach that can provide a solution from a technical perspective but at the same time demands ethical considerations is the emerging possibility wherein companion animals with cancer become potential subjects for new technology assessments [193]. An example can be found in in dogs with naturally acquired tumors [194]. Together, it seems likely that both small- and large-animal evaluations are needed to best prepare an image guidance technology for clinical evaluation. Such evaluations, combined with toxicity testing, will gather the evidence needed to apply for ethical approval for first-in-human evaluations.

Clinically surgical tasks are divided according to anatomy, disease indication and even the type of surgical intervention. This helps ensure expertise for surgeons and helps create some form of quality assurance from a healthcare perspective. From a technological perspective, however, most of these boundaries seem irrelevant. Physical and technical factors (e.g., open vs laparoscopic, soft tissue vs rigid anatomies) drives the design of new technologies. That said, most chemical and engineering efforts mentioned above still find applications in multiple settings. The success stories in the field of image-guided surgery are based on technologies that maximally align with innovations made in other fields. For example, initial work on fluorescence laparoscopy presented in prostate cancer surgery [195] was later transferred to breast surgery [196] and the technique is now also implemented during i.e., laparoscopic colorectal surgery [94, 197]. Such knowledge sharing can be considered highly valuable as it helps boost innovation across disciplines.

## Conclusion

With all the promising technologies being developed under the umbrella of image-guided surgery, it remains essential to maintain a helicopter view of this highly multidisciplinary and rapidly expanding field. Hereby, we need to make sure that the clinical needs remain aligned with tracer chemistry, device physics and the increasing digitization of the operating room.

## Supplementary Information

**Additional file 1.** 

## Data Availability

Not applicable.

## Abbreviations

US: Ultrasound
CT: Computed tomography
MRI: Magnetic resonance imaging
PET: Positron emission tomography
PSMA: Prostate specific membrane antigen
ICG: Indocyanine green
3D: Three-dimensional
SPECT: Single photon emission computed tomography
OR: Operating room
