# Supplementary material for: The current status and future prospects for molecular imaging-guided precision surgery
Source: Cancer Imaging. 2022 Sep 6;22:48. doi: 10.1186/s40644-022-00482-2 (PMC9446692; doi:10.1186/s40644-022-00482-2)
Supplement: Supplementary file 1 — Additional file 1. [file 40644_2022_482_MOESM1_ESM.docx]

**Point-by-point rebuttal to the reviewer comments**

We would like to thank the reviewers for their comments and suggestions, which we have adapted accordingly (resulting adaptations in the manuscript have been marked in red in the accompanying manuscript file). Please find our response to the reviewers below.

Reviewer #1: The manuscript entitled "Trends and prospects in image guided precision surgery" described image guided technologies for surgery.
I am not sure why the authors put the title "Trends and prospects in image guided precision surgery".

Response: Our intention was to provide an overview of the field of image-guided surgery over the years and include the current status and future prospects. To avoid any confusion the title of the manuscript was adapted to “The current status and future prospects for molecular imaging-guided precision surgery”.

This is a review article; all they did was a literature search of the MEDLINE/Pubmed, Scopus and Web of Science database. Over 60% of references are outdated.

Response: As an overview over time is given, we intentionally included references of the first application of a particular technique, which indeed are “older” references. We have reviewed the reference list and updated this where required. Also, additional references have been added. As this has affected the manner of search and selection, this section was omitted from the manuscript.

Did not we have any advanced technology development during 1990-now? Only intraoperative MRI in 1994 (Figure 1)?

Response: We have adapted the title of Figure 1 to “Timeline of the introduction of image-guided surgical technologies” to clarify that this Figure focusses on the first introduction of particular image-guidance techniques. Further technological advancement is discussed thereafter in the text.

Moreover, most of the figures presented are not informative.

Response: We have tried to provide figures that match the broad content of the manuscript. If the reviewer has suggestion for adaptation, we are of course we are willing to adapt the figures accordingly.

Overall, it was a poorly constructed manuscript and really does not cover the recent advancement of imaging technology for surgery.

Response: The manuscript was edited extensively.

Reviewer #2: In this manuscript, the authors try to review trends and prospects in image guided precision surgery. The manuscript is not well written, totally confuse, contains a lot of totally incorrect statements and is also full of typos. Listing all the errors, misunderstandings and misconceptions would be a waste of time.

Response: We apologize to the reviewer and have made extensive adaptations to the manuscript in order to improve the readability.

Reviewer #3: The Authors wrote a comprehensive review on image guided surgery. The paper is timely and well written.

Response: We thank the reviewer for their positive response.
